# Guizhi Fuling pill attenuates liver fibrosis *in vitro* and *in vivo* via inhibiting TGF-β1/Smad2/3 and activating IFN-γ/Smad7 signaling pathways

**DOI:** 10.1080/21655979.2022.2054224

**Published:** 2022-04-07

**Authors:** Zhongliang Liu, Baogui Xu, Yaping Ding, Xianjun Ding, Zuisu Yang

**Affiliations:** a Department of Oncology, Zhoushan Hospital of Traditional Chinese Medicine (Affiliated to Zhejiang University of Traditional Chinese Medicine), Zhoushan, P.R. China; b School of Food and Pharmacy, Zhejiang Ocean University, Zhoushan, China; c Department of Nutrition, Zhoushan Hospital of Traditional Chinese Medicine (Affiliated to Zhejiang University of Traditional Chinese Medicine), Zhoushan, P.R. China; d Department of Infectious Diseases, Zhoushan Hospital of Traditional Chinese Medicine (Affiliated to Zhejiang University of Traditional Chinese Medicine), Zhoushan, P.R. China; e Department of Infectious Diseases, Zhoushan Hospital, P.R. China

**Keywords:** Chronic liver diseases, liver fibrosis, guizhi fuling pill, TGF‐β1/smad2/3, CUG-binding protein 1

## Abstract

Liver fibrosis resulting from chronic liver injuries (CLI) is a common health problem globally. Guizhi Fuling pill (GZFL), a modern preparation from traditional Chinese medicine, exhibited anti-dysmenorrhea, anti-inflammatory, and immune-regulative effects. However, the effect of GZFL on liver fibrosis remains unknown. In this research, LX-2 cells were stimulated with acetaldehyde for mimicking liver fibrosis progression *in vitro*. In addition, carbon tetrachloride (CCl_4_)-induced mouse model of liver fibrosis was established as well. The data revealed GZFL obviously suppressed the proliferation and triggered the apoptosis of acetaldehyde-stimulated LX-2 cells. In addition, GZFL prevented acetaldehyde-induced activation of LX-2 cells via downregulation of TGF-β1, p-Smad2, p-Smad3, CUGBP1, and upregulation of p-STAT1 and Smad7. Meanwhile, GZFL significantly alleviated CCl_4_‑induced liver fibrosis, as evidenced by the decrease of ALT and AST levels. Moreover, GZFL downregulated the expressions of TGF-β1, p-Smad2, p-Smad3, and CUGBP1 in CCl_4_-treated mice. Furthermore, GZFL remarkably elevated the levels of IFN-γ, p-STAT1, and Smad7 in CCl_4_-treated mice. To sum up, GZFL was able to inhibit liver fibrosis *in vitro* and *in vivo* through suppressing TGF-β1/Smad2/3-CUGBP1 signaling and activating IFN-γ/STAT1/Smad7 signaling. Thus, GZFL might have a potential to act as a therapeutic agent for anti-fibrotic therapy.

## Graphical Abstract

**Figure:**
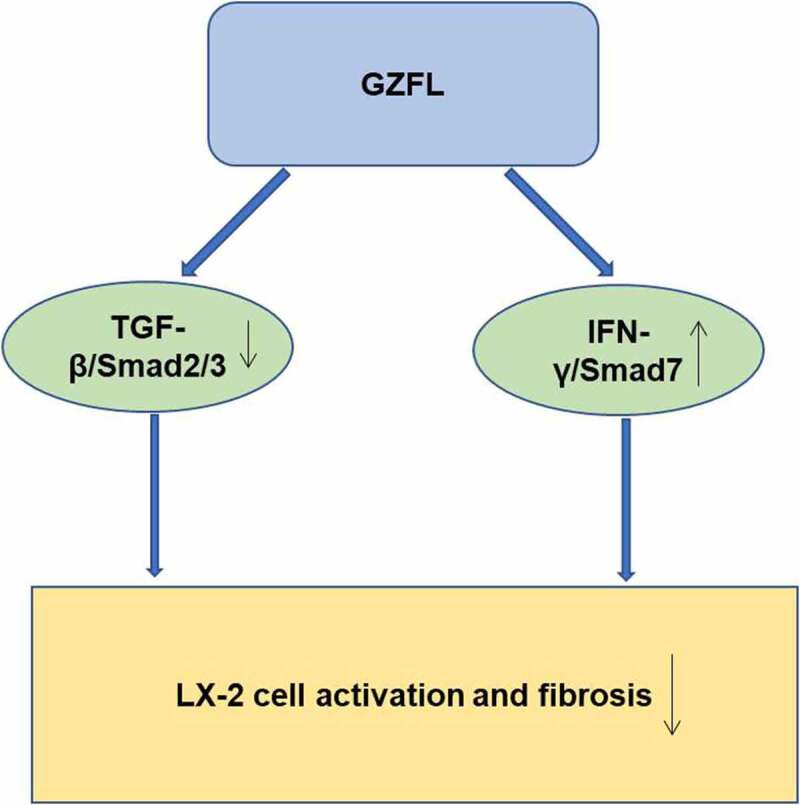

## Introduction

Chronic liver diseases (CLD) have become a major disorder worldwide, which is characterized by sustained inflammatory response and fibrogenesis[1]. Liver fibrosis is a wound-healing response to chronic liver injuries (CLI) that occurs in various types of CLD [2]. CLD including hepatitis, nonalcoholic steatohepatitis, alcoholic fatty liver, and cholestasis could lead to liver fibrosis, and liver fibrosis may ultimately evolve into liver cirrhosis even hepatocellular carcinoma [3–6]. Meanwhile, there are about 18 million new cases of liver fibrosis per year in China [7]. At present, obethicolic acid has been approved for the treatment of liver fibrosis, while the outcomes remain limited. Thus, exploration of novel effective anti-fibrotic drugs for patients with liver fibrosis is extremely necessary.

Liver fibrosis is characterized by increased accumulation of extracellular matrix (ECM) proteins including type I collagen (collagen I) [8,9]. In addition, hepatic stellate cells (HSCs) play a major role in hepatic fibrogenesis[10]. HSCs activated by inflammation was able to convert to myofibroblast-like cells and then generate excessive ECM, eventually leading to hepatic fibrosis [11,12]. Evidence has shown that acetaldehyde, a main toxic metabolite of alcohol, could trigger liver fibrosis via HSCs[13]. Acetaldehyde is able to stimulate the activation of HSCs by promoting transforming growth factor‐β1 (TGF‐β1) synthesis in cells[14]. In addition, acetaldehyde could increase the expressions of collagens and smooth muscle α‐action (α‐SMA) in HSCs[14].

It has been reported that Chinese herbal compounds exerted anti-fibrotic effects, including Guizhi Fuling pill (GZFL)[15]. GZFL is a Chinese herbal compound composed of five medicinal herbs, including *Cinnamomi Ramulus, Poria, Moutan Cortex, Persicae Semen*, and *Paeoniae Radix Alba*[16]. GZFL exhibits various pharmacological properties including anti-dysmenorrhea, anti-inflammatory, and immunoloregulation effects [16–18]. In addition, Lian et al. found that a heteropolysaccharide from *Moutan Cortex* could alleviate tubulointerstitial fibrosis in a rat with diabetic nephropathy[19]. Zhang et al. found that amygdalin, a component from *Semen Persicae*, was able to attenuate pancreatic fibrosis in a rat with chronic pancreatitis[20]. Moreover, Bushen Yijing Decoction (BSYJ) exerts an anti-fibrotic effect via regulating MicroRNA-26a/FLI1 axis[21]. However, the detailed function of GZFL in liver fibrosis remains unexplored. Based on the above backgrounds, we hypothesized that GZFL might have the potential in the treatment of liver fibrosis. Hence, we explored the effects of GZFL on liver fibrosis in the current study.

Since HSC activation could lead to the progression of liver fibrosis[22], the effect of GZFL on the viability of HSCs should be assessed. Additionally, the inflammatory responses and the activation of TGF-β signaling could cause the occurrence of liver fibrosis[23]. Moreover, the data of liver function was important for the diagnosis of liver fibrosis[24]. Thus, the related bioassays were required in this study. As expected, the results confirmed that GZFL could inhibit the progression of liver fibrosis, and this research might shed new lights on exploring new strategies against liver fibrosis.

## Materials and methods

### Cell culture and Materials

LX-2 cell line was obtained from the Xiangya Central Experiment Laboratory, Central South University (Changsha, China). Cells were cultured in DMEM (Thermo Fisher Scientific, Waltham, MA, USA) containing 10% fetal bovine serum (FBS, Gibco, Waltham, MA, USA), 100 mg/ml streptomycin, and 100 U/ml penicillin at 37°C in a humidified atmosphere with 5% CO_2_. LX-2 cells were treated with acetaldehyde (400 μM; Macklin Inc., Shanghai, China) for 24 h, and then treated with colchicine (Sigma Aldrich, St. Louis, MO, USA) or GZFL (Chengdu Jiuzhitang Jinding Pharmaceutical Co., Ltd., Chengdu, China) for another 24 h. The procedure was performed in accordance with the previous reference[15].

## Cell viability assay

LX-2 cells (5 × 10^3^ cells) were plated onto 96-well plates overnight. Then, cells were treated with GZFL (0, 1, 2, 4, 6, 8, 10, 25, or 50 mg/ml) or colchicine (0, 1, 2, 4, 8, 16, 32, or 64 μg/ml) for 24 h. After that, cell-counting kit 8 (CCK-8) reagent (10 μL; Dojindo, Shanghai, China) was added into each well for 2 h. Subsequently, the absorbance was recorded using a SpectraMax M2 microplate reader (Molecular Devices, San Jose, CA, USA) at 450 nm. The procedure was in accordance with the previous reference[25].

## AO/EB staining assay

LX-2 cells were stained with the dye mixture (1:1) of acridine orange (AO; 100 μg/ml) and ethidium bromide (EB; 100 μg/ml) for 5 min in darkness. Later on, cells were photographed with a fluorescent microscope (Nikon, Tokyo, Japan). The procedure was conducted in accordance with the previous reference[26].

## Hochest33342/PI staining assay

LX-2 cells were fixed for 15 min in 4% paraformaldehyde. After that, cells were stained with Hochest33342 (10 μl, 100 mg/ml) for 15 min and then stained with PI (5 μl, 1 g/l) for 30 min. Later on, cells were observed using a fluorescent microscope[27].

## Western blot assay

Each group of proteins (30 μg/lane) were separated by 10% SDS-PAGE and transferred to a PVDF membrane. Then, the membrane was incubated with primary antibodies against α-SMA (1:5000; Proteintech, Wuhan, China), Collagen I (1:8000; Proteintech), p-STAT1 (1:1000; Affinity biosciences, Cambridge, UK), STAT1 (1:5000; Proteintech), CUG-binding protein 1 (CUGBP1; 1:2000; Proteintech), TGF-β1 (1:1000; Proteintech), TGFBR2 (1:1000; Affinity biosciences), Smad1 (1:5000; Proteintech), p-Smad2 (1:1000; Affinity biosciences), Smad2 (1:5000; Proteintech), p-Smad3 (1:1000; Affinity biosciences), Smad3 (1:3000; Proteintech), Smad7 (1:1000; Proteintech), Bax (1:1000; Affinity biosciences), active caspase 3 (1:1000; Affinity biosciences), BCL-2 (1:1000; Proteintech), active caspase 9 (1:1000; Affinity biosciences), and GAPDH (1:10,000; Proteintech) overnight at 4°C. Later on, the membrane was probed with the secondary antibody for 1 h. Subsequently, the blots were developed using an electrochemiluminescence reagent (Thermo Fisher Scientific). The procedure was conducted in accordance with the previous reference[28].

## Animal study

Male ICR mice (4–6‐week age) were obtained from Zhejiang Provincial Experimental Animal Center. All animal experiments were approved by the Ethics Committee of Experimental Animal Ethics Committee of Zhejiang Ocean University (Zhoushan, China) (Permission No. 2020–0026) and performed according to the procedures of National Institutes of Health guide for the care and use of laboratory animals. Animals were allowed to adapt to their environment for 7 days. After that, animals were divided into five groups randomly: control, CCl_4_, CCl_4_ + colchicine, CCl_4_ + low dose GZFL (L-GZFL), CCl_4_ + high dose GZFL (H-GZFL) groups (n = 5). In the control group, mice were intraperitoneally treated with saline (0.9% NaCl) for 4 weeks (twice per week). Mice in the CCL_4_, CCl_4_ + colchicine, CCl_4_ + L-GZFL or CCl_4_ + H-GZFL groups were intraperitoneally administrated with CCl_4_ solution (3 ml/kg, dissolved in soybean oil) for 4 weeks (twice per week). After 4 weeks of treatment, mice in the control and CCl_4_ groups were given 0.9% saline by gastric gavage per day for another 4 weeks. Meanwhile, mice in the CCl_4_ + colchicine, CCl_4_ + L-GZFL or CCl_4_ + H-GZFL groups were given colchicine (0.1 mg/kg), L-GZFL (125 mg/kg) or H-GZFL (250 mg/kg) three times a day by gastric gavage for another 4 weeks, respectively. The drug administration was in line with previous described[29]. The body weights of mice were recorded weekly. All mice were sacrificed at 9 weeks, and the liver and spleen weights of mice were recorded. Liver or spleen coefficient = liver or spleen weight/body weight.

## Histological evaluation

Liver samples were sectioned to a thickness of 5 μm. Subsequently, slides were subjected to H&E staining and Masson’s trichrome staining as previously described[30]. Next, the injury of liver tissue was observed using an Olympus BH2 microscope.

## ELISA assay

The levels of alanine aminotransferase (ALT), aspartate aminotransferase (AST), total bilirubin (T-BIL), albumin (ALB), alkaline phosphatase (AKP), glutathione sulfotransferase (GSH-ST) in serum of mice were detected using ELISA kits. In addition, the concentrations of hydroxyproline (Hyp), malondialdehyde (MDA), superoxide dismutase (SOD), glutathione peroxidase (GSH-Px), total antioxidant capacity (T-AOC), catalase (CAT), hyaluronic acid (HA) and laminin (LN), type III procollagen (PC III) and type IV collagen (Col-IV) in the liver tissues of mice were detected using ELISA kits. These ELISA kits were obtained from Nanjing Jiancheng Bioengineering Institute (Nanjing, China).

Meanwhile, the concentrations of IL-6, IFN-γ, IL-1β, TNF-α in the liver tissues of mice were detected by ELISA kits (Elabscience Biotechnology Co., Ltd., Wuhan, China).

## RT-qPCR assay

Total RNA was isolated from liver tissues using the TRIzol reagent and transcribed using a HiScript 1st Strand cDNA Synthesis Kit (Vazyme, Piscataway, NJ, USA). After that, qPCR was carried out using AceQ qPCR SYBR Green Master Mix (without ROX) (Vazyme). Gene expression was normalized against GAPDH and calculated using 2^−ΔΔct^ method[31]. The sequences of primers were as follows: TGF-βR2 forward, 5’-AAACUACUAGGUAAAGGCACUUUU-3’ and reverse, 5’-GATAAAUUUAAAGCUCUGUGCC-3’; Smad2 forward, 5’-GGAGGACTGAGAAGGTGAGGC-3’ and reverse, 5’-GGCAAGGGGACATCCTCTG-3’; Smad3 forward, 5’-CTCTTCTCATTCCTGCTTG-3’ and reverse, 5’-CTCCACTTGGTGGTTTGT-3’; α-SMA forward, 5’-CACAGAAGGAGTGGCTAA-3’ and reverse, 5’-CCATAACGCACTAGGTTT-3’; GAPDH forward, 5’-GTCCACCGCAAATGCTTCTA-3’ and reverse, 5’-TGCTGTCACCTTCACCGTTC-3’.

## Statistical analysis

Each experiment was repeated independently at least three times. Data are presented as the mean ± standard deviation (S.D.) The statistical significance of differences was calculated by One-way analysis of variance (ANOVA) and Tukey’s tests. P values of <0.05 were considered as statistically significant.

## Results

### GZFL inhibits acetaldehyde-induced LX-2 cells activation by regulating cell proliferation and apoptosis

**Figure 1.**
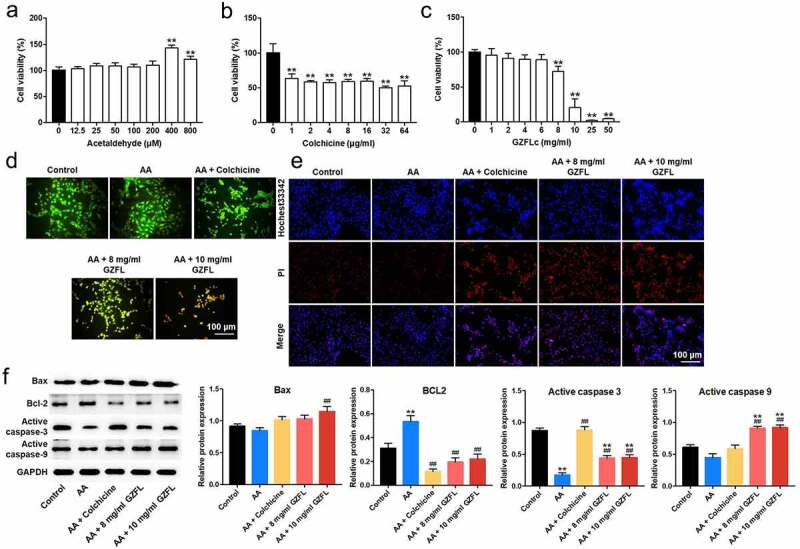
**GZFL inhibits acetaldehyde-induced LX-2 cells activation through regulating cell viability and apoptosis**. (a) LX-2 cells were treated with different concentrations of acetaldehyde and cell viability was detected with CCK-8 assay (b) LX-2 cells were treated with different concentrations of colchicine and cell viability was detected with CCK-8 assay. (c) LX-2 cells were treated with different concentrations of GZFL and cell viability was detected with CCK-8 assay. (d, e) LX-2 cells were stimulated with 400 μM for 24 h, and then treated with colchicine (4 μg/ml) or GZFL (8 or 10 mg/ml) for another 24 h. AO/EB and Hochest3342/PI staining assays were performed to determine cell apoptosis. (f) Western blot was used to determine Bax, BCL-2, active caspase 3 and active caspase 9 expressions in LX-2 cells. **P < 0.01 vs. control group; ^##^P < 0.01 vs. AA group; n = 3.

In order to explore the effect of GZFL on liver fibrosis *in vitro*, LX-2 cells were activated with acetaldehyde firstly. Then, the effect of acetaldehyde on LX-2 cell viability was evaluated. The result showed that 400 μM acetaldehyde remarkably increased the viability of LX-2 cells (Figure 1a). In addition, colchicine (4 μg/ml), used as a positive control, induced about 50% growth inhibition in LX-2 cells (Figure 1b). Moreover, 8 and 10 mg/ml GZFL markedly decreased the proliferation of LX-2 cells (Figure 1c). Thus, GZFL at 8 or 10 mg/ml dose was used for the subsequent *in vitro* experiments.

Next, AO/EB staining and Hochest3342 staining indicated that 8 or 10 mg/ml GZFL obviously induced the apoptosis in acetaldehyde-stimulated LX-2 cells (Figure 1d and 1E). Meanwhile, acetaldehyde significantly reduced the expression of active caspase 3 and upregulated BCL2 expression in LX-2 cells, whereas the effects of acetaldehyde were partially reversed by GZFL treatments (figure 1f). To sum up, GZFL could suppress acetaldehyde-induced LX-2 cells activation by regulating cell proliferation and apoptosis.

### GZFL inhibits acetaldehyde-induced LX-2 cells activation through suppressing TGF-β1/Smad2/3 signaling and activating IFN-γ/STAT1/Smad7 pathway

**Figure 2.**
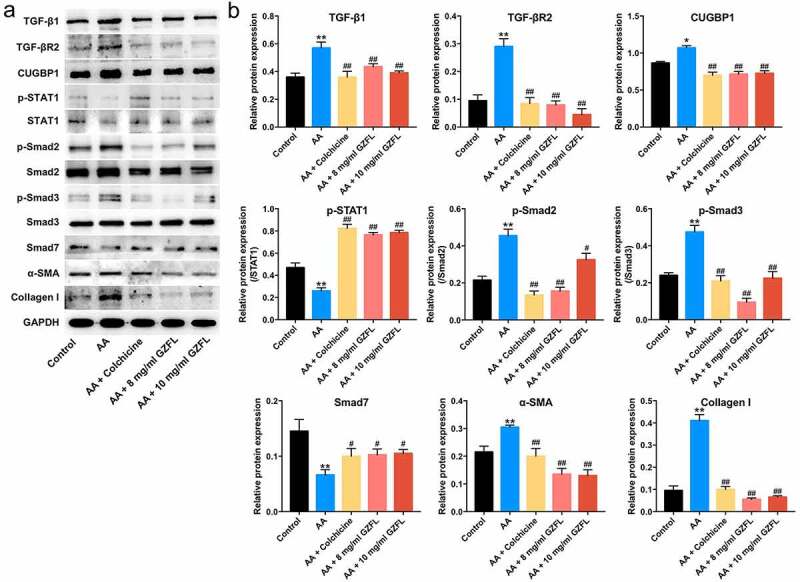
**GZFL inhibits acetaldehyde-induced LX-2 cells activation through suppressing TGF-β1/Smad2/3 signaling and activating IFN-γ/STAT1/Smad7 signaling**. LX-2 cells were exposed to acetaldehyde (AA; 400 μM) for 24 h, followed by exposure to colchicine (4 μg/ml) or GZFL (8 or 10 mg/ml) for another 24 h. (a, b) TGF-β1, TGF-βR2, CUGBP1, p-STAT1, p-Smad2, p-Smad3, Smad7, α-SMA and Collagen I expressions in LX-2 cells were detected by western blot assay. *P < 0.05, **P < 0.01 vs. control group; ^#^P < 0.05, ^##^P < 0.01 vs. AA group; n = 3.

Evidences have shown that TGF-β1/Smad2/3 signaling play a pro-fibrotic role, while IFN-γ/STAT1/Smad7 signaling pathway exhibit an anti-fibrotic role in liver fibrosis progression [32,33]. Importantly, CUGBP1 has been emerged as a key molecule in liver fibrosis, which may affect the pro- and anti-fibrotic signaling pathways[34]. Therefore, we investigated the effects of GZFL on TGF-β1/Smad2/3, IFN-γ/STAT1 or CUGBP1 signaling in acetaldehyde-treated LX-2 cells. The data indicated that GZFL notably decreased the expressions of TGF-β1, TGF-βR2, CUGBP1, p-Smad2, p-Smad3, α-SMA and Collagen I and upregulated the expressions of p-STAT1 and Smad7 in acetaldehyde-treated LX-2 cells (Figure 2a and 2B). Furthermore, no significant differences in IFN-γ levels were detected between acetaldehyde and GZFL treatment groups (Supplementary Fig. 1). These data suggested that GZFL could inhibit acetaldehyde-induced LX-2 cells activation through suppressing TGF-β1/Smad2/3, CUGBP1 signaling and activating IFN-γ/STAT1/Smad7 pathway.

### GZFL ameliorates CCl_4_-induced liver fibrosis in mouse in vivo

**Figure 3.**
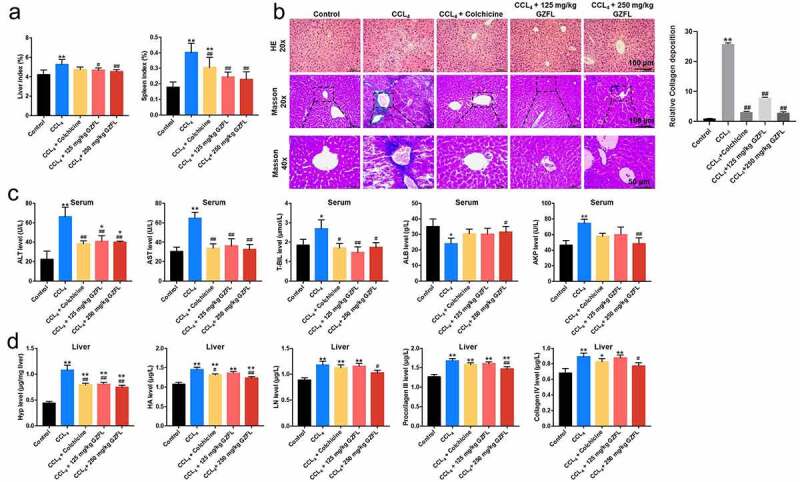
**GZFL ameliorates CCl_4_-induced liver fibrosis *in vivo***. (a) The liver and spleen index in mice with CCl_4_-induced liver fibrosis. (b) Analysis of liver injury in liver tissues with HE staining. Analysis of collagen deposition in liver tissues with Masson’s trichrome staining assay. (c) ALT, AST, T-BIL, ALB and AKP levels in serum samples in mice with CCl_4_-induced liver fibrosis were measured with ELISA assay. (d) The levels of liver fibrotic markers Hyp, HA, LN, PC III and Col-IV in liver tissues in mice with CCl_4_-induced liver fibrosis were measured with ELISA assay. *P < 0.05, **P < 0.01 vs. control group; ^#^P < 0.05, ^##^P < 0.01 vs. CCL_4_ group; n = 5.

To further explore the role of GZFL in liver fibrosis *in vivo*, CCl_4_-induced mouse model of liver fibrosis was constructed. As shown in Figure 3a, mice subjected to intraperitoneal injection with CCl_4_ displayed an increase of liver or spleen index, while these phenomena were reversed by GZFL treatment. Furthermore, HE staining assay revealed that mice subjected to CCl_4_ displayed obvious liver injury (unordered liver structure and localized injury and necrosis); however, CCl_4_-induced liver injury were completely reversed by GZFL treatment (Figure 3b). Moreover, masson’s trichrome staining revealed a higher accumulation of collagen in CCl_4_-treated mice compared to control group; whereas GZFL treatment greatly attenuated these pathologic changes (Figure 3b).

Next, the mouse liver function was determined by analyzing serum ALT, AST, and T-BIL, ALB and AKP levels. As indicated in Figure 3c, CCl_4_ significantly elevated serum ALT, AST, T-BIL and AKP levels and reduced ALB levels in mouse; however, these changes were all reversed by GZFL treatments. Meanwhile, GZFL treatment notably downregulated the levels of liver fibrotic markers Hyp, HA, LN, PC III and Col-IV in CCl_4_-treated mice (Figure 3d). Collectively, GZFL was able to ameliorate CCl4-induced liver fibrosis in mouse *in vivo*.

### *GZFL ameliorates CCl_4_-induced liver fibrosis in mouse* in vivo *via inhibiting inflammation and oxidative stress*

**Figure 4.**
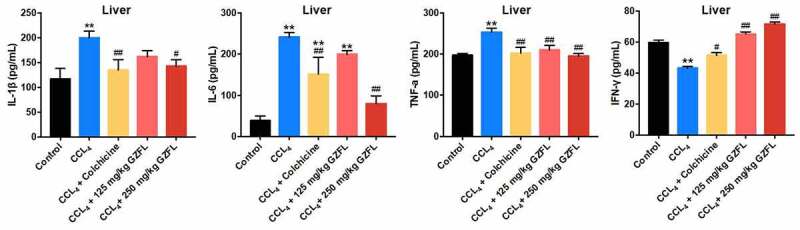
**GZFL ameliorates CCl_4_-induced liver fibrosis *in vivo* via exerting anti-inflammation effect**. The levels of IL-1β, IL-6, TNF-α, IFN-γ in liver tissues of CCl_4_-treated mice were measured with ELISA assay. **P < 0.01 vs. control group; ^#^P < 0.05, ^##^P < 0.01 vs. CCL_4_ group; n = 5.

**Figure 5.**
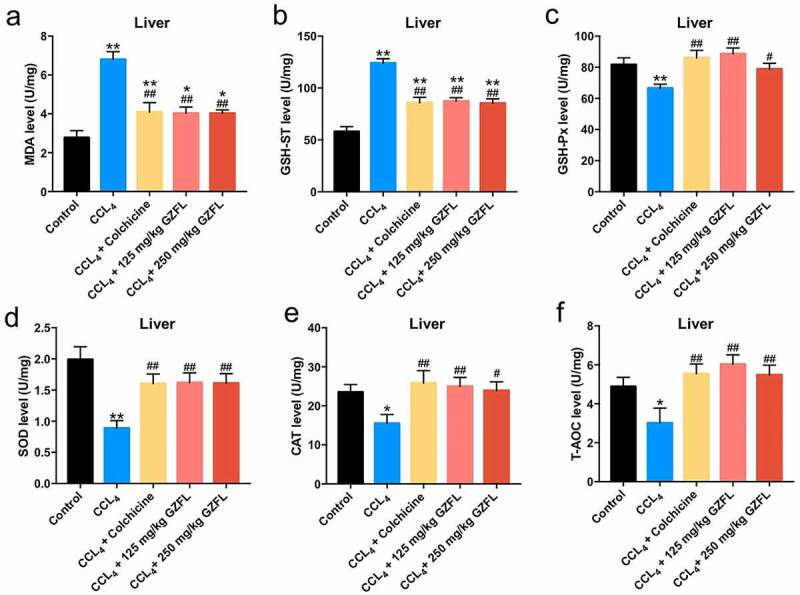
**GZFL ameliorates CCl_4_-induced liver fibrosis *in vivo* via exerting anti-oxidation effect. (A, B, C, D, E, F)** The levels of MDA, GSH-ST, GSH-Px, SOD, CAT, T-AOC in liver tissues of CCl_4_-treated mice were measured with ELISA assay. *P < 0.05, **P < 0.01 vs. control group; ^#^P < 0.05, ^##^P < 0.01 vs. CCL_4_ group; n = 5.

With the purpose of exploring whether GZFL could exhibit anti-inflammatory and anti-oxidative effects in CCl_4_-treated mice, ELISA assay was applied. We found that GZFL treatment markedly decreased the levels of IL-1β, IL-6, TNF-α and elevated the expression of IFN-γ in the liver tissues of CCl_4_-treated mice (Figure 4). Meanwhile, GZFL treatment notably reduced MDA and GSH-ST and upregulated GSH-Px, SOD, CAT, and T-AOC levels in the liver tissues of CCl_4_-treated mice (Figure 5a, 5B, 5C, 5D, 5E, and 5 F). To sum up, GZFL could ameliorate CCl_4_-induced liver fibrosis in mouse *in vivo* via exerting anti-inflammatory and anti-oxidative activities.

### *GZFL ameliorates CCl_4_-induced liver fibrosis in mouse* in vivo *by inhibiting TGF-β1/Smad2/3 signaling and activating IFN-γ/STAT1 signaling*

**Figure 6.**
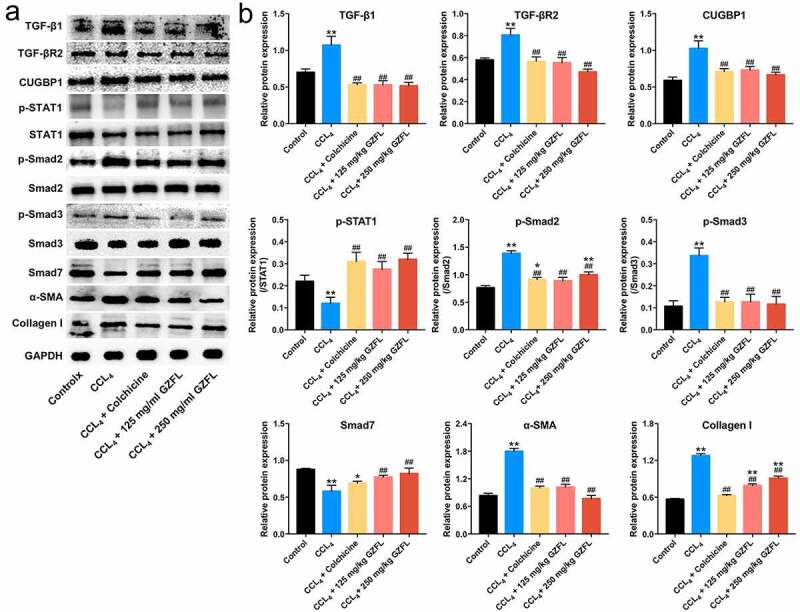
**GZFL ameliorates CCl_4_-induced liver fibrosis *in vivo* by inhibiting TGF-β1/Smad2/3 signaling and activating IFN-γ/STAT1/Smad7 signaling**. (a, b) TGF-β1, TGF-βR2, CUGBP1, p-STAT1, p-Smad2, p-Smad3, Smad7, α-SMA and Collagen I expressions in liver tissues of CCl_4_-treated mice were detected using western blot assay. *P < 0.05, **P < 0.01 vs. control group; ^##^P < 0.01 vs. CCL_4_ group; n = 5.

We finally explored the effects of GZFL on TGF-β1/Smad2/3 signaling and IFN-γ/STAT1 signaling in CCl_4_-induced mouse model of liver fibrosis by using western blot. As shown in Figure 6a and 6B and Supplementary Fig. 2A-2D, TGF-β1, TGF-βR2, CUGBP1, p-Smad2, p-Smad3, Collagen I and α-SMA levels were remarkably elevated and p-STAT1 and Smad7 levels were reduced in liver tissues of CCl_4_-treated mice; whereas these changes were reversed by GZFL treatment. Collectively, GZFL could reduce CCl_4_-induced liver fibrosis *in vivo* by inhibiting TGF-β1/Smad2/3, CUGBP1 signaling and activating IFN-γ/STAT1/Smad7 signaling.

## Discussion

Liver fibrosis, a serious health problem worldwide, is the common pathological basis of chronic liver diseases [35,36]. Currently, there are no effective therapies for liver fibrosis. Fortunately, Traditional Chinese medicine has attracted more attention for their anti-fibrosis effects [37,38]. Hsu et al. found that *Graptopetalum paraguayense* could inhibit liver fibrosis in diethylnitrosamine-induced rat liver injury model via suppressing TGF-β signaling[39]. Chinese herbal formula Fuzheng Huayu was able to alleviate liver fibrosis in CCl_4_-treated rats[40]. However, the role of GZFL in liver fibrosis remains unclear.

The activated HSCs, main producers of ECM, contribute to the formation of liver fibrosis [23,41]. Acetaldehyde was able to trigger the activation of HSCs via inducing cell proliferation, which is accompanied by the increase of Collagen I and α-SMA[14]. In this study, we found GZFL treatments significantly reversed acetaldehyde-induced LX-2 cells activation. Furthermore, GZFL obviously induced the apoptosis of acetaldehyde-stimulated LX-2 cells. Meng et al. reported that carvedilol could inhibit HSCs activation via promoting cell apoptosis[42]. Consistent with previous reports, GZFL was able to inhibit acetaldehyde-induced LX-2 cells activation through suppressing cell proliferation and promoting apoptosis.

Furthermore, we found that CCl_4_ notably induced liver injury and fibrosis in mice, as determined by the elevated levels of ALT and AST. Meanwhile, CCl_4_ upregulate liver fibrotic markers HA, LN, PC III, and Col-IV in mice. However, GZFL treatments significantly alleviated liver injury and fibrosis in CCl_4_-treated mice. In addition, we found that GZFL exhibited anti-inflammation and anti-oxidation effects in CCl_4_-treated mice. Consistently, Feriani et al. showed that *zygophyllum album* leaves extract alleviated liver fibrosis in deltamethrin-treated rats via improving liver function and inhibiting inflammation and oxidative stress[43]. These data showed that GZFL could ameliorate liver fibrosis *in vivo* via exerting anti-inflammatory and anti-oxidative activities.

Evidences have shown that TGF-β could trigger HSCs activation and fibrotic injury; suppressing TGF-β signaling could alleviate the liver fibrosis process [41,44]. As we know, TGF-β promotes fibrogenesis via activating Smad2 and Smad3, which is negatively regulated by Smad7[23]. Chen et al indicated that Baihe Wuyao decoction could reduce liver fibrosis in CCl_4_-treated mice through inhibiting TGF-β1/Smads signaling[45]. Our data indicated that GZFL remarkably decreased the levels of TGF-β1, TGF-βR2, p-Smad2, p-Smad3 and increased the level of Smad7 *in vitro* and *in vivo*. All these data indicated that GZFL was able to ameliorate liver fibrosis *in vitro* and *in vivo* though suppressing TGF-β1/Smad2/3 signaling pathway.

It has been shown that IFN-γ/STAT1 signaling exhibits anti-fibrotic activity in liver cells [33,46]. IFN-γ could elevate Smad7 expression through activation of STAT-1 signaling, thereby inhibiting TGF-β signaling[33]. Jeong et al. showed that STAT1 could alleviate liver fibrosis by inhibition of TGF-β signaling[47]. In this study, GZFL elevated IFN-γ, p-STAT1, and Smad7 expressions in liver tissues of CCl_4_-treated mice. These data showed that GZFL was able to ameliorate liver fibrosis by activating IFN-γ/STAT1/Smad7 signaling.

RNA‐binding protein CUGBP1 is a key molecule in regulating liver injury [48,49]. Recently, Wu et al. revealed that TGF-β could elevate the CUGBP1 expression in HSCs[34]. In addition, CUGBP1 promoted HSCs activation via downregulating IFN-γ levels[50]. These data suggested that CUGBP1 is a key molecule in liver fibrosis, which mediated the crosstalk between TGF-β1/Smad2/3 and IFN-γ/STAT1 signaling pathway. The present results showed that GZFL notably decreased the levels of TGF-β1 and CUGBP1 and upregulated the level of IFN-γ in liver tissues of CCl_4_-treated mice. Thus, we deduced GZFL decreased the expression of CUGBP1, and then triggered IFN-γ/Smad7 signaling in activated HSCs. In another word, the pro-fibrogenic activity of TGF-β was limited by GZFL. All these data suggested that GZFL was able to ameliorate liver fibrosis *in vitro* and *in vivo* by modulating the crosstalk between TGF-β1/Smad2/3 and IFN-γ/STAT1/Smad7 signaling pathway via CUGBP1.

## Conclusion

GZFL could inhibit acetaldehyde‑induced cellular fibrosis and alleviate CCL_4_‑induced liver fibrosis by inhibiting TGF-β1/Smad2/3, CUGBP1 signaling and activating IFN-γ/STAT1/Smad7 signaling. Therefore, GZFL might have a potential to act as a therapeutic agent for anti-fibrotic therapy.

## Supplementary Material

Supplemental Material

## Data Availability

The datasets used and/or analyzed during the current study are available from the corresponding author on reasonable request.
