# Supplementary figures and images for: Guizhi Fuling pill attenuates liver fibrosis in vitro and in vivo via inhibiting TGF-β1/Smad2/3 and activating IFN-γ/Smad7 signaling pathways
Source: Bioengineered. 2022 Apr 7;13(4):9357–68. doi: 10.1080/21655979.2022.2054224 (PMC9161976; doi:10.1080/21655979.2022.2054224)

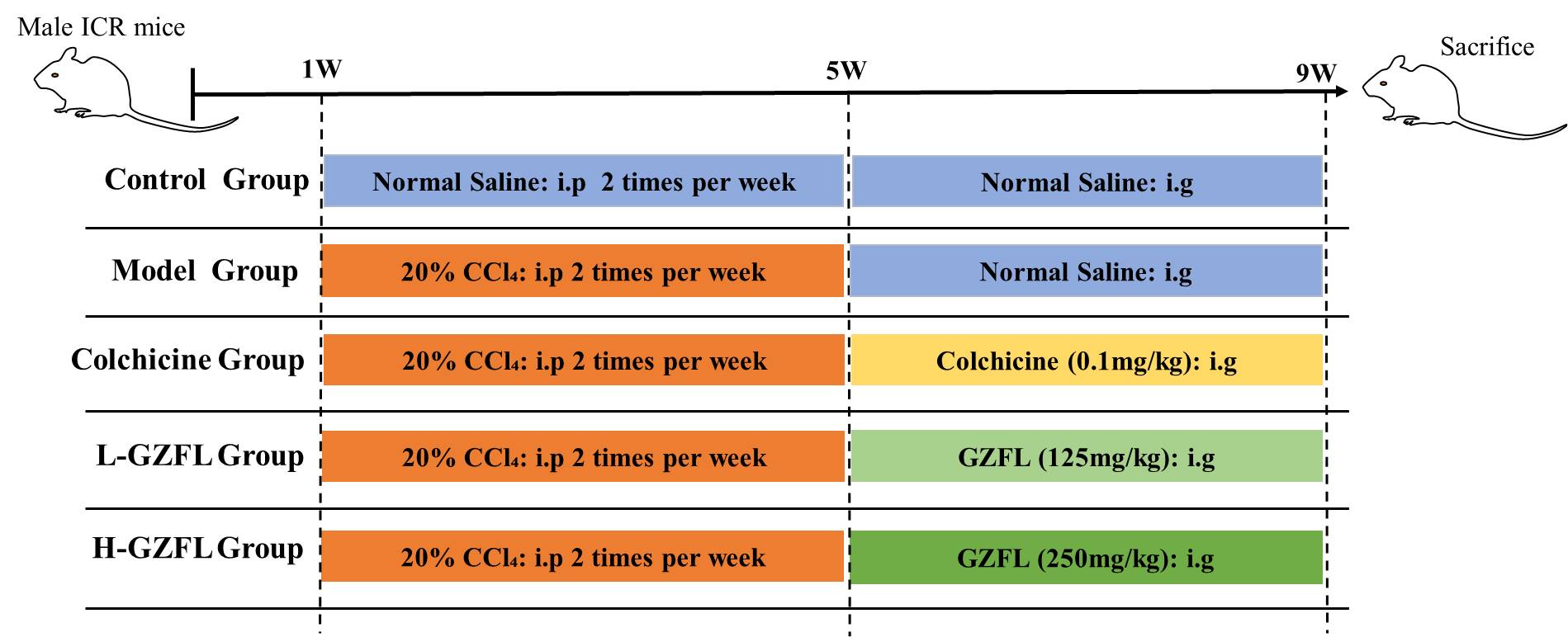

Supplement: Supplemental Material [file KBIE_A_2054224_SM2925.zip › supplementary/Experimental design.jpg]

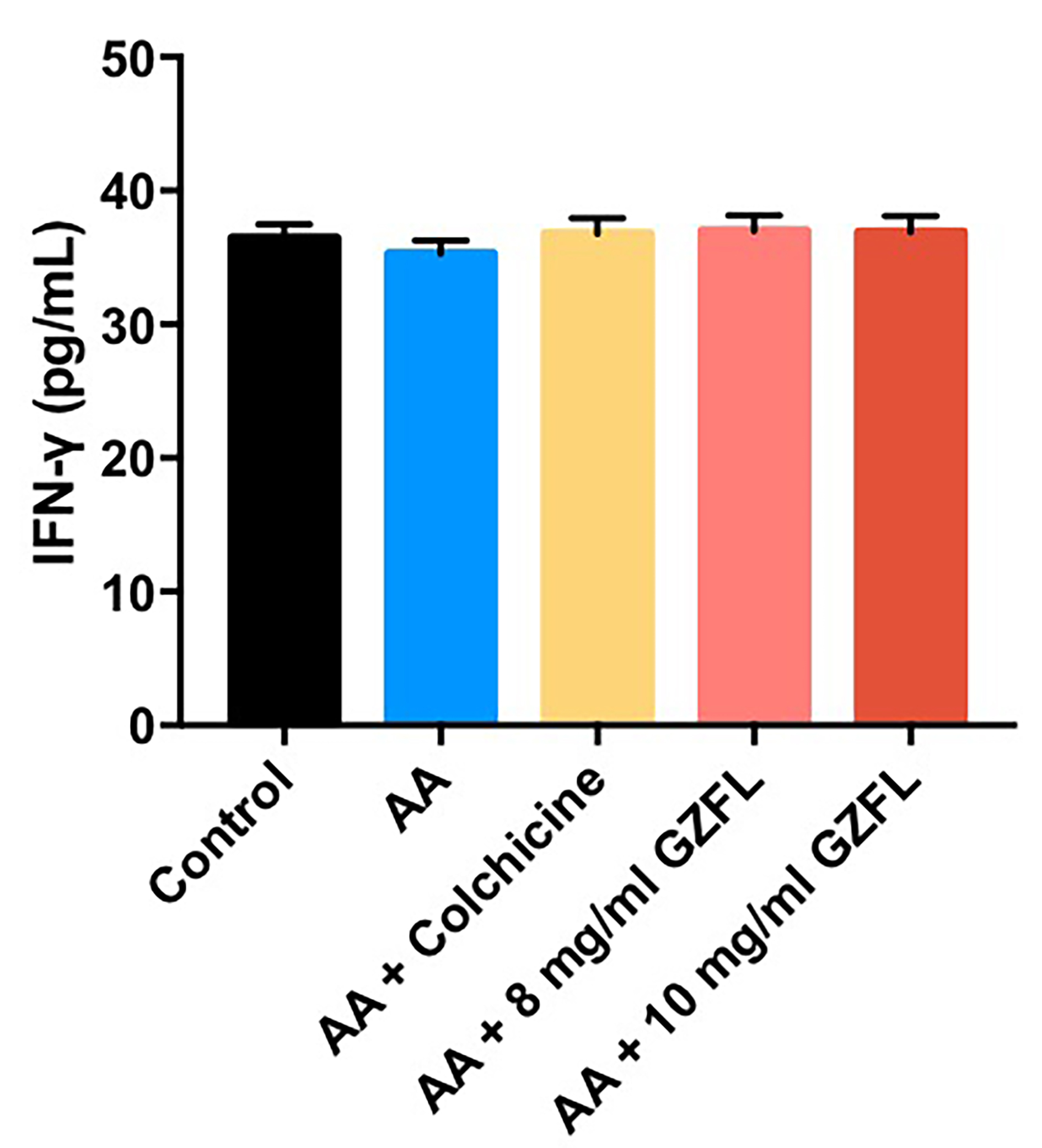

Supplement: Supplemental Material [file KBIE_A_2054224_SM2925.zip › supplementary/Supplementary figure1.jpg]

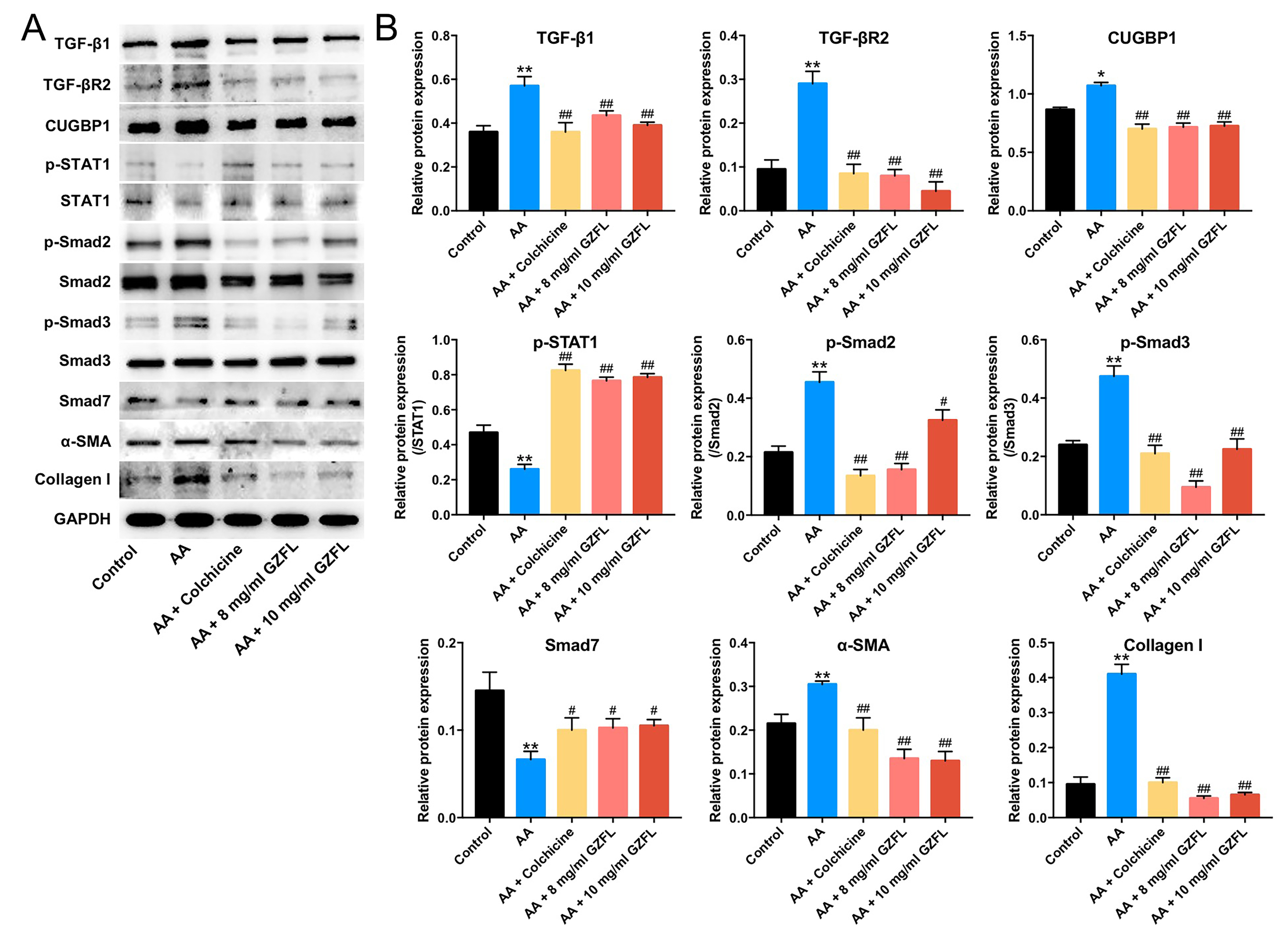

Supplement: Supplemental Material [file KBIE_A_2054224_SM2925.zip › supplementary/Supplementary figure2.jpg]
