# Supplementary material for: Guizhi Fuling pill attenuates liver fibrosis in vitro and in vivo via inhibiting TGF-β1/Smad2/3 and activating IFN-γ/Smad7 signaling pathways
Source: Bioengineered. 2022 Apr 7;13(4):9357–68. doi: 10.1080/21655979.2022.2054224 (PMC9161976; doi:10.1080/21655979.2022.2054224)

6/10/2013, 18:32; Size: 696x520; Exp: 210008ms; Bin: 2x2; Modif: No; Disp BWG: (35832, 65532, 0.38)
File: Smad7 5.31-1.tif at: C:\Users\洪涛\Documents\Tencent Files\704877645\FileRecv\LX-2细胞WB\TGF-Smads信号通路\Smad7

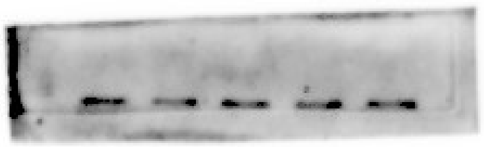

Supplement: Supplemental Material [file KBIE_A_2054224_SM2925.zip › supplementary/western blot raw data.zip!/western blot raw data/Figure 2/Smad7.pdf]
