# Supplementary material for: Guizhi Fuling pill attenuates liver fibrosis in vitro and in vivo via inhibiting TGF-β1/Smad2/3 and activating IFN-γ/Smad7 signaling pathways
Source: Bioengineered. 2022 Apr 7;13(4):9357–68. doi: 10.1080/21655979.2022.2054224 (PMC9161976; doi:10.1080/21655979.2022.2054224)

8/24/2021, 20:52; Size: 696x520; Exp: n/a; Bin: 1x1; Modif: No; Disp BWG: (56137, 65534, 1.00)
File: SMA 6.7(用）.tif at: C:\Users\洪涛\Documents\Tencent Files\704877645\FileRecv\LX-2细胞WB\标志蛋白\SMA

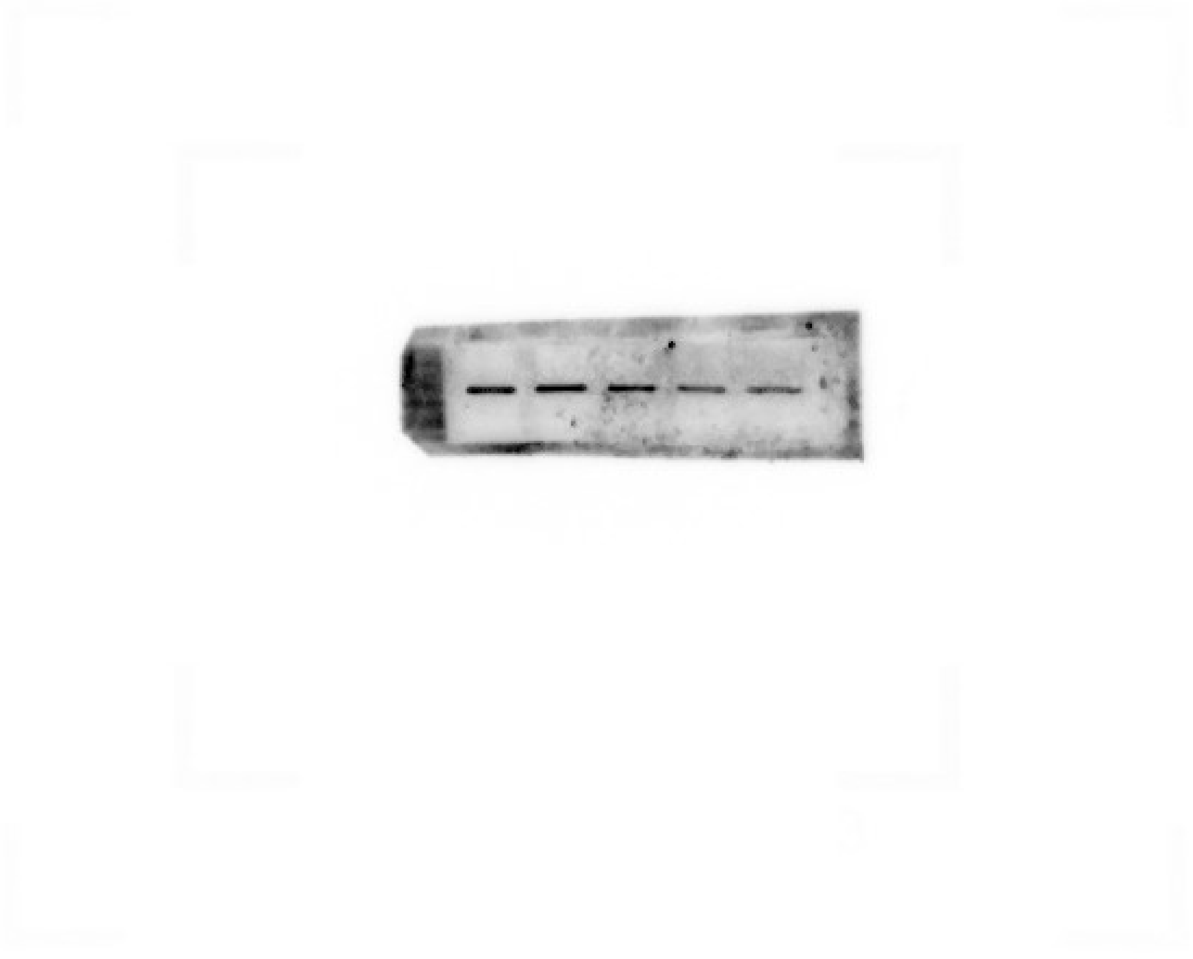

Supplement: Supplemental Material [file KBIE_A_2054224_SM2925.zip › supplementary/western blot raw data.zip!/western blot raw data/Figure 2/a-SMA.pdf]
