# Supplementary material for: Guizhi Fuling pill attenuates liver fibrosis in vitro and in vivo via inhibiting TGF-β1/Smad2/3 and activating IFN-γ/Smad7 signaling pathways
Source: Bioengineered. 2022 Apr 7;13(4):9357–68. doi: 10.1080/21655979.2022.2054224 (PMC9161976; doi:10.1080/21655979.2022.2054224)

6/10/2013, 15:33; Size: 696x520; Exp: 60008ms; Bin: 2x2; Modif: No; Disp BWG: (16492, 65532, 0.45)
File: LX-2 p-STAT1 10.28-1.tif at: C:\Users\洪涛\Documents\Tencent Files\704877645\FileRecv\10月LX-2细胞WB\10月LX-2细胞WB

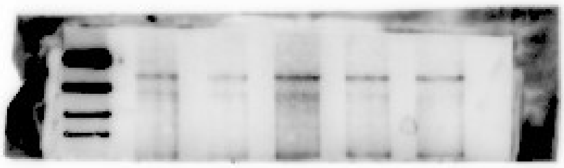

Supplement: Supplemental Material [file KBIE_A_2054224_SM2925.zip › supplementary/western blot raw data.zip!/western blot raw data/Figure 2/p-STAT1.pdf]
