# Supplementary material for: Guizhi Fuling pill attenuates liver fibrosis in vitro and in vivo via inhibiting TGF-β1/Smad2/3 and activating IFN-γ/Smad7 signaling pathways
Source: Bioengineered. 2022 Apr 7;13(4):9357–68. doi: 10.1080/21655979.2022.2054224 (PMC9161976; doi:10.1080/21655979.2022.2054224)

6/10/2013, 15:53; Size: 696x520; Exp: n/a; Bin: 1x1; Modif: No; Disp BWG: (62263, 65534, 0.45)
File: p-Smad3 6.6（用）.tif at: C:\Users\洪涛\Documents\Tencent Files\704877645\FileRecv\LX-2细胞WB\TGF-Smads信号通路\Smad3和P-Smad3

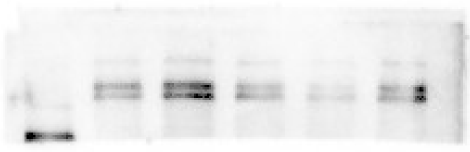

Supplement: Supplemental Material [file KBIE_A_2054224_SM2925.zip › supplementary/western blot raw data.zip!/western blot raw data/Figure 2/p-Smad3.pdf]
