# Supplementary material for: Guizhi Fuling pill attenuates liver fibrosis in vitro and in vivo via inhibiting TGF-β1/Smad2/3 and activating IFN-γ/Smad7 signaling pathways
Source: Bioengineered. 2022 Apr 7;13(4):9357–68. doi: 10.1080/21655979.2022.2054224 (PMC9161976; doi:10.1080/21655979.2022.2054224)

6/10/2013, 14:42; Size: 696x520; Exp: 30008ms; Bin: 2x2; Modif: No; Disp BWG: (62866, 65533, 0.45)
File: a-SMA 3.17.tif at: C:\Users\洪涛\Documents\Tencent Files\704877645\FileRecv\肝组织WB\肝-肝纤标志蛋白\a-SMA

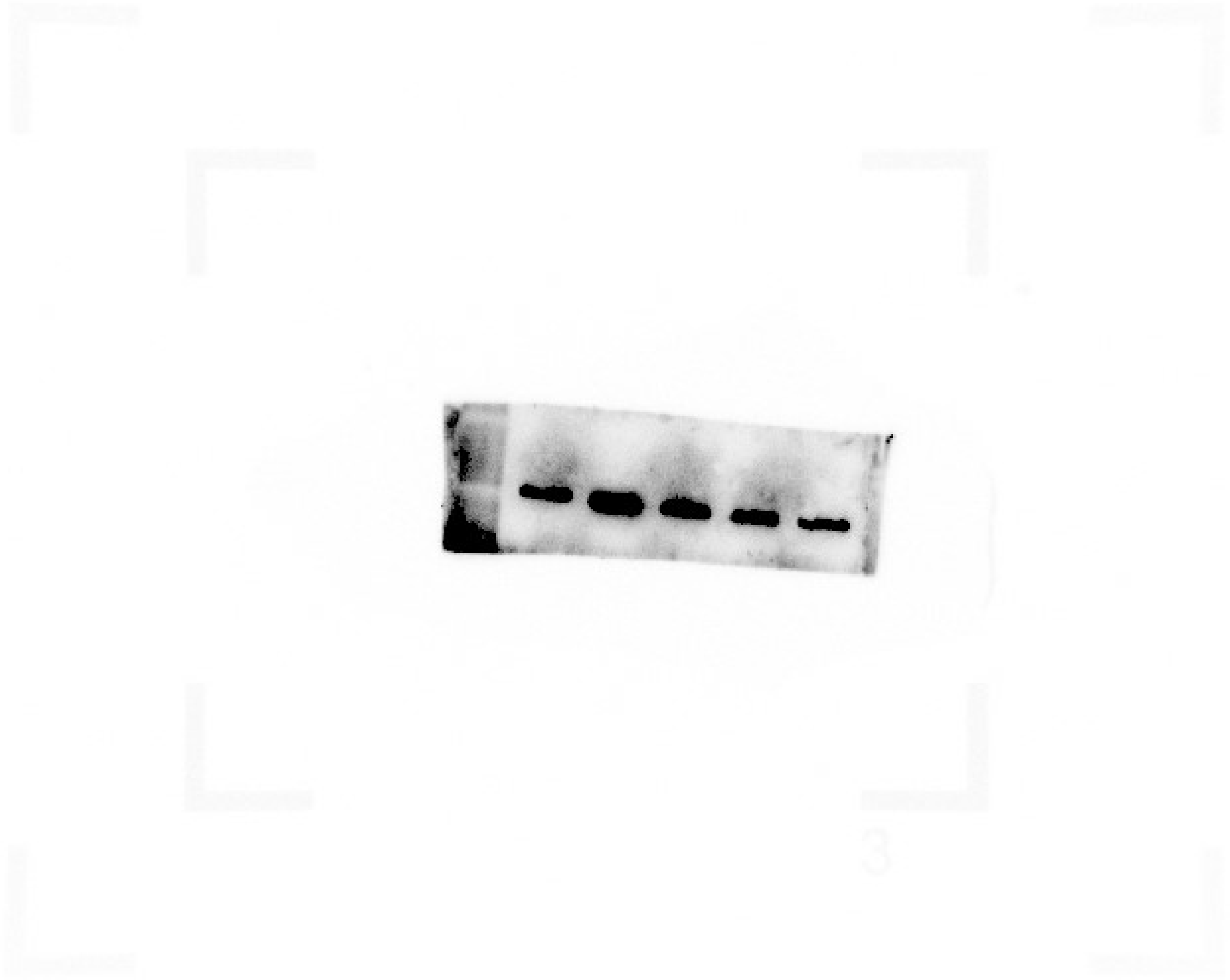

Supplement: Supplemental Material [file KBIE_A_2054224_SM2925.zip › supplementary/western blot raw data.zip!/western blot raw data/Figure 6/a-SMA.pdf]
